# Impact of exposure time in awake prone positioning on clinical outcomes of patients with COVID-19-related acute respiratory failure treated with high-flow nasal oxygen: a multicenter cohort study

**DOI:** 10.1186/s13054-021-03881-2

**Published:** 2022-01-07

**Authors:** Mariano Esperatti, Marina Busico, Nora Angélica Fuentes, Adrian Gallardo, Javier Osatnik, Alejandra Vitali, Elizabeth Gisele Wasinger, Matías Olmos, Jorgelina Quintana, Santiago Nicolas Saavedra, Ana Inés Lagazio, Facundo Juan Andrada, Hiromi Kakisu, Nahuel Esteban Romano, Agustin Matarrese, Mariela Adriana Mogadouro, Giuliana Mast, Claudia Navarro Moreno, Greta Dennise Rebaza Niquin, Veronica Barbaresi, Alejandro Bruhn Cruz, Bruno Leonel Ferreyro, Antoni Torres, Argentine Collaborative Group on High Flow and Prone Positioning, Anabel Miranda Tirado, María Constanza Viñas, Juan Manuel Pintos, Maria Eugenia Gonzalez, Maite Mateos, Mariela Marisol Laiz, Jose Garcia Urrutia, Micaela Ruiz Seifert, Emilce Mastroberti

**Affiliations:** 1 Intensive Care Unit, Hospital Privado de Comunidad, Mar del Plata, Argentina; 2 Escuela Superior de Medicina, Universidad Nacional de Mar del Plata, Mar del Plata, Argentina; 3 Intensive Care Unit, Clínica Olivos SMG, Av. Maipú 1660, B1602 ABQ, Olivos, Provincia de Buenos Aires Argentina; 4 Sociedad Argentina de Terapia Intensiva, Buenos Aires, Argentina; 5 Intensive Care Unit, Sanatorio Clínica Modelo de Morón, Morón, Buenos Aires Argentina; 6 Universidad de Morón, Morón, Buenos Aires Argentina; 7 Intensive Care Unit, Hospital Aleman, Ciudad Autónoma de Buenos Aires, Argentina; 8 Universidad del Salvador, Buenos Aires, Argentina; 9 Intensive Care Unit, Sanatorio de La Trinidad Palermo, Ciudad Autónoma de Buenos Aires, Argentina; 10 Intensive Care Unit, Hospital Universitario Austral, Pilar, Buenos Aires Argentina; 11 Universidad Austral, Pilar, Buenos Aires Argentina; 12 Departement of Intensive Medicine, Pontificia Universidad Católica de Chile, Santiago, Chile; 13 Interdepartmental Division of Critical Care Medicine, University of Toronto, Toronto, Canada; 14 Pulmonary Department, Hospital Clinic, Universitat de Barcelona, IDIBAPS, CIBERES, Barcelona, Spain

**Keywords:** Acute respiratory failure, Awake, COVID-19, Endotracheal intubation, Mortality, Prone position

## Abstract

**Background:**

In patients with COVID-19-related acute respiratory failure (ARF), awake prone positioning (AW-PP) reduces the need for intubation in patients treated with high-flow nasal oxygen (HFNO). However, the effects of different exposure times on clinical outcomes remain unclear. We evaluated the effect of AW-PP on the risk of endotracheal intubation and in-hospital mortality in patients with COVID-19-related ARF treated with HFNO and analyzed the effects of different exposure times to AW-PP.

**Methods:**

This multicenter prospective cohort study in six ICUs of 6 centers in Argentine consecutively included patients > 18 years of age with confirmed COVID-19-related ARF requiring HFNO from June 2020 to January 2021. In the primary analysis, the main exposure was awake prone positioning for at least 6 h/day, compared to non-prone positioning (NON-PP). In the sensitivity analysis, exposure was based on the number of hours receiving AW-PP. Inverse probability weighting–propensity score (IPW-PS) was used to adjust the conditional probability of treatment assignment. The primary outcome was endotracheal intubation (ETI); and the secondary outcome was hospital mortality.

**Results:**

During the study period, 580 patients were screened and 335 were included; 187 (56%) tolerated AW-PP for [median (p25–75)] 12 (9–16) h/day and 148 (44%) served as controls. The IPW–propensity analysis showed standardized differences < 0.1 in all the variables assessed. After adjusting for other confounders, the OR (95% CI) for ETI in the AW-PP group was 0.36 (0.2–0.7), with a progressive reduction in OR as the exposure to AW-PP increased. The adjusted OR (95% CI) for hospital mortality in the AW-PP group ≥ 6 h/day was 0.47 (0.19–1.31). The exposure to prone positioning ≥ 8 h/d resulted in a further reduction in OR [0.37 (0.17–0.8)].

**Conclusion:**

In the study population, AW-PP for ≥ 6 h/day reduced the risk of endotracheal intubation, and exposure ≥ 8 h/d reduced the risk of hospital mortality.

**Supplementary Information:**

The online version contains supplementary material available at 10.1186/s13054-021-03881-2.

## Background

Severe acute respiratory syndrome coronavirus 2 (SARS-CoV-2) infection is responsible for causing coronavirus disease 2019 (COVID-19) [1]. While most patients are asymptomatic or mildly symptomatic, a subset develops acute respiratory failure and acute respiratory distress syndrome (ARDS) [2]. The use of invasive mechanical ventilation for the treatment of these conditions is associated with high mortality rates, with reports exceeding 50% in both developed and developing countries [3, 4]. Given this high mortality risk reported, there is a high value in identifying strategies that can mitigate the progression of lung injury and prevent the need for invasive ventilation [5, 6].

In patients with acute respiratory failure (ARF), the use of high-flow nasal oxygen therapy (HFNO) has been found to decrease the need for endotracheal intubation and mortality when compared to conventional oxygen therapy and noninvasive ventilation [7, 8]. Thus, it has been increasingly used to support respiratory failure in patients with COVID-19 during the pandemic [6]. Prone positioning has consistently been shown to reduce mortality in patients with moderate to severe ARDS receiving invasive mechanical ventilation [9, 10]. However, the effect of prone positioning on clinical outcomes in patients not receiving invasive mechanical ventilation remains unclear. This intervention has been applied to spontaneously breathing patients with COVID-19-related ARF [11]. The combination of HFNO and awake prone position (AW-PP) has been described to be associated with a low rate of endotracheal intubation (ETI) in case series of patients with COVID-19 [11–13]. These findings were confirmed in a recent meta-trial that compared HFNO plus AW-PP versus HFNO plus usual care [14]. However, the effects of exposure times in AW-PP on relevant clinical outcomes remain unclear.

We evaluated the effect of prone positioning on the risk of endotracheal intubation and in-hospital mortality in a cohort of patients admitted to ICU with COVID-19-related acute respiratory failure initially treated with high-flow nasal oxygen and analyzed the effects of different exposure times to AW-PP.

## Methods

### Design and scope of the study

We conducted a prospective multicenter cohort study at 6 ICUs of 6 centers in Argentina. Four of these were University Affiliated Centers described in the Additional File. The study was conducted between June 2020 and January 2021, corresponding to the first wave in Argentina, and is reported following guidelines from Strengthening the Reporting of Observational studies in Epidemiology (STROBE) [15]. The Internal Review Board from the 6 centers approved the study including waived informed consent (Universidad Austral Comité Institucional de Evaluación N°20-072; Comité de Ética en Investigación SATI for Trinidad Palermo; Comité de Docencia e Investigación Sanatorio Clínica Modelo de Morón; Comité de Ética e Investigación Hospital Alemán; Consejo Institucional de Revisión de Estudios de Investigación Hospital Privado de Comunidad Nº 2919/2143/2020; Comité de Ética en Investigación Clínica Olivos). The interventions carried out were part of the usual practice at each center, and researchers guaranteed confidentiality of their patient’s information.

### Study population

We included patients older than 17 years admitted to the ICU with a confirmed diagnosis of COVID-19 (real-time PCR) and receiving HFNO for at least 4 h. Patients received HFNO when any of the following criteria were present: (a) peripheral oxygen saturation (SpO_2_) < 92% with oxygen > 4 L/min; (b) increased work of breathing with use of accessory respiratory muscles, and a respiratory rate > 30/min; (c) PaO_2_/FiO_2_ratio < 200 mmHg. Patients with respiratory failure secondary to a different etiology, decreased level of consciousness, presence of shock requiring vasopressors, immediate need for intubation, use of positive-pressure ventilation prior to HFNO, and with do-not-intubate orders were excluded.

### Study procedures

Immediately after admission to the ICU, the inclusion criteria regarding oxygenation and/or work of breathing were checked. Then, a high-flow nasal cannula sized according to the nares size (Optiflow, Fisher and Paykel Healthcare) was placed and connected to a specific device for the provision of high-flow O_2_ (Humidoflo HF-2900, GGM Co., Taiwan; or AIRVO, Fisher and Paykel Healthcare, Auckland, New Zealand) or to an ICU respirator in high flow mode (Neumovent GraphNet Advance, Tecme S.A., Córdoba, Argentina; or Monnal T75, Air Liquide Medical System, France). The initial flow was 50–70 L/min, with the FiO_2_ necessary to obtain an SpO_2_ > 92%. No maximum FiO_2_ limits were established. Once therapy with HFNO was started, participants were encouraged and assisted by the healthcare team to rotate from supine to prone position for as long as possible, taking breaks for personal hygiene and eating. No maximum time limits for prone position were established. Patients who tolerated HFNO for the next 4 h were included in the study. Where prone positioning was not tolerated by patients, they were assisted to remain in the lateral position, alternating right and left decubitus. These interventions were maintained during the study period until one of the following criteria was met: maintenance of SpO_2_ > 92% with FiO_2_ ≤ 40%, and flow ≤ 40 L/min for a period > 12 h in the supine position; or endotracheal intubation. Once clinical stability and gas stability were attained with HFNO, flow and oxygen were progressively weaned. The de-escalation was initiated with a gradual FiO_2_ decrease until ≤ 40% was reached. Subsequently, the flow level was progressively reduced until reaching ≤ 40 L/min. Once these requirements were met, a low-flow nasal cannula was placed with an O_2_ flow of 3–5 L/min in the supine position. Analgesic drugs (opioids, paracetamol) or light sedation (dexmedetomidine) was allowed and indicated according to the criteria of the healthcare team. (For more details about the procedures, see Additional file 1.)

### Variables and measurements

We collected data on patients’ demographics (age, sex, body mass index [BMI]), comorbidities, severity scores upon ICU admission (APACHE II and SOFA), chronology of the disease (time from the onset of symptoms to hospital admission and ICU admission), vital signs, laboratory parameters, Respiratory rate Oxygenation index (ROX index) [16], and chest computed tomography score (CT score) determined as the sum of lung involvement, ranging from 0—no involvement to 25—maximum involvement [17].

The main exposure of this study was awake prone positioning. For analysis purposes, we defined awake prone positioning as remaining in this position for at least 6 h per day, based on recent evidence suggesting that this is the minimum time that could impact ETI rate [11]. We also collected information on the number of hours in prone, and the number of days with at least 6 h of prone position per patient. The following data were recorded: (a) the predominant position adopted by the patient, defined as the position in which the patient spent most h/day, i.e., prone, lateral or supine position; (b) the average number of h/day in that position; and (c) the number of days of exposure to said position for a period of ≥ 6 h/day (AW-PP). Patients who did not receive prone positioning for at least 6 h served as controls. Since AW-PP exposure was initially attempted in the total population of patients included, some patients in the control group could have had some degree of prone exposure (< 6 h/d).

The primary outcome was defined as the receipt of endotracheal intubation. The decision to intubate was based on the criteria of the attending healthcare team. However, intubation was recommended for anyone meeting the following criteria: deterioration of neurologic status, hemodynamic instability, or if two or more of the following criteria were met: decline in oxygen saturation with SpO_2_ < 90% for more than 5 min (not explained by technical failures), lack of improvement in the signs of respiratory muscle fatigue, impossibility to control airway secretions and respiratory acidosis with pH < 7.30 [7]. The secondary outcome was hospital mortality. Additionally, we described other clinical outcomes: ICU and hospital length of stay, time to endotracheal intubation, and severe complications related to AW-PP.

### Statistical analysis

We used descriptive statistics to describe patients’ baseline characteristics. Standardized mean differences were used to assess the balance between baseline characteristics of patients who received prone positioning and controls. A standardized mean difference of 10% or less was considered to indicate good balance [18].

Since treatment allocation was not randomly assigned, inverse probability of treatment weighting (IPW) was used to control for potential confounding by indication (Additional file 1: Table E8). The IPW is an extension of the propensity score method used to summarize the conditional probability of treatment assignment [19, 20]. Based on subject matter knowledge and previous literature, we used a direct acyclic graph (DAG) to identify and select variables potentially associated with both awake prone positioning and study outcomes [21, 22] (Additional File 1: Fig. E3). First, we created a propensity score by fitting a multivariable logistic regression model with awake prone positioning as the binary outcome. The following potential confounders were included in the model: age, sex, BMI, comorbidities, smoking status, SOFA and APACHE II scores, days from symptoms onset to hospital admission, oxygen therapy previous to ICU admission (days and administration mode), antibiotic therapy, corticosteroid therapy, ROX index, TC score, C-reactive protein (CRP), and use of light sedation. Once the propensity score was created, each patient was assigned a weight which was inversely proportional to the probability of being treated as estimated by the propensity score. These weights were stabilized and trimmed by removing observations with extreme weights (i.e., percentile < 1% and > 99%) [23]. Finally, the primary analysis was performed by fitting a logistic regression model with awake prone position as the main exposure and endotracheal intubation as the dependent variable. This model was fitted using the weighted population by the previous procedure. Moreover, we included in the model the participant center and the chronological time since the onset of the pandemic as independent variables (double robust approach). Measures of association are expressed as odds ratios (OR) with 95% confidence intervals (CI), which were created using bootstrapping to account for the correlation of the weighted population [23].

The dynamics of the pandemic and the number of patients admitted to ICUs could not be predicted. Considering the lack of data on the effectiveness of the intervention, as many patients as possible were consecutively recruited without a predefined sample size.

Several post hoc sensitivity analyses were conducted in order to assess the robustness of our findings. First, we recategorized the exposure based on the number of hours receiving awake prone positioning (≥ 8, ≥ 12 and ≥ 16 h/d). Second, we performed restricted analyses according to hypoxemia severity (PaO_2_/FiO_2_ < 150 mmHg and < 100 mmHg) and the predominant body position (prone, lateral or supine). Finally, we performed a restricted analysis comparing AW-PP > 6 h versus patients with “zero” hours in prone position.

Given the likelihood of an unmeasured confounder, we estimated the *e* value as a way to determine how strong the association between such unmeasured confounder with both the exposure and outcome should be to fully explain the estimated effect [24].

Every test was two-sided, and a *p* value < 0.05 was considered statistically significant. All analyses were performed with STATA version 15.1. For more details about statistical analysis, see Additional file 1.

## Results

**Fig. 1:**
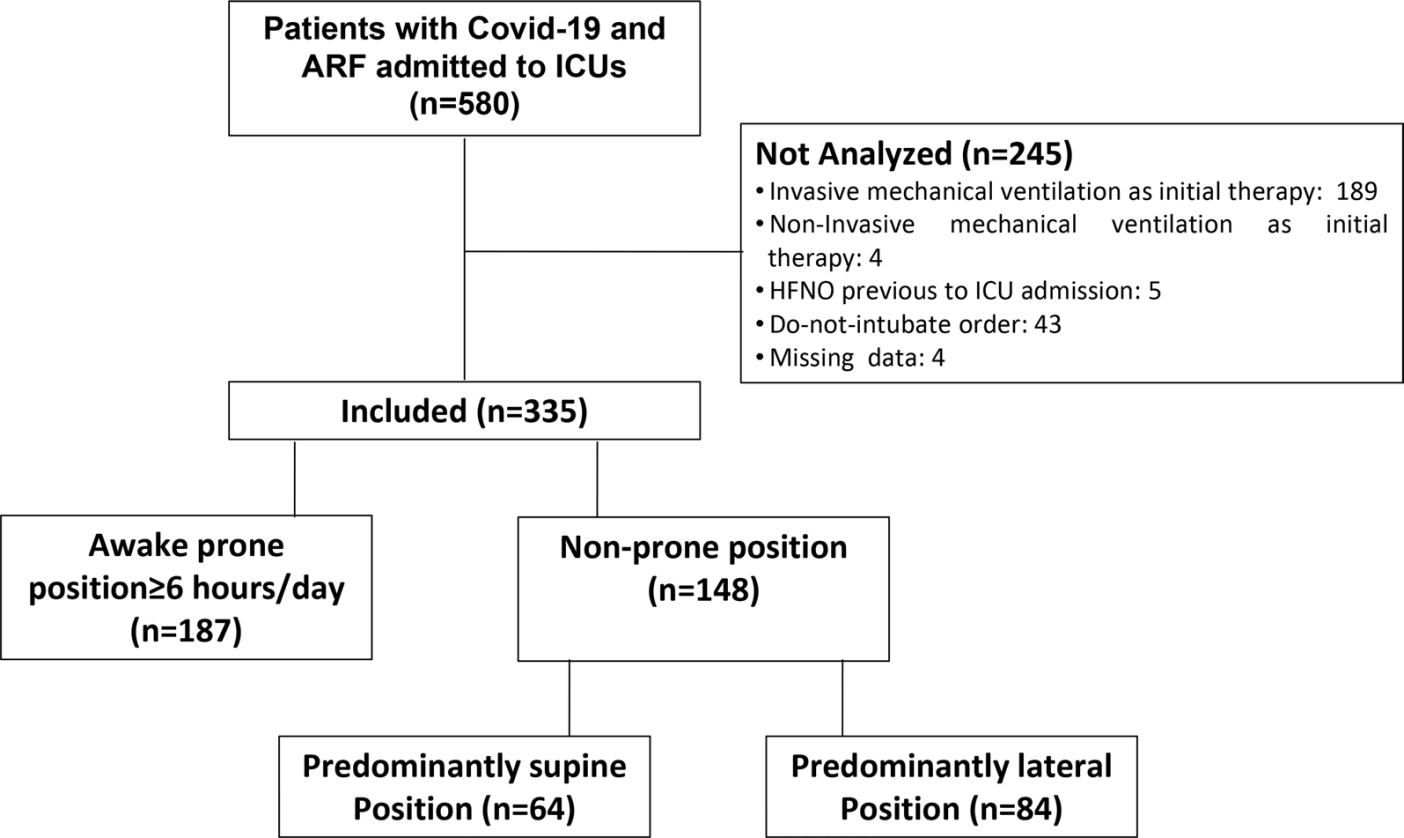
Flowchart of the cohort. Abbreviations. ARF: acute respiratory failure. ICU: intensive care unit

During the study period, 580 patients with COVID-19-related ARF were admitted to the participating ICUs (Fig. 1). Three hundred thirty-five patients met the inclusion criteria and were included in the analysis, of which 187 (56%) were treated with HFNO and ≥ 6 h/day of AW-PP (intervention group) and 148 (44%) were treated with HFNO but did not complete 6 h of awake prone position, and therefore served as the control group (NON-PP). Patients in the AW-PP group spent a median (p25–75) of 12 (9–16) h/day in the prone position, during 5 (3–8) days. In the control group, 84 patients (25%) remained predominantly in the lateral position for a median (p25–75) of 8 (6–10) hours and 3.5 (2–6) days; and 64 patients (19%) remained predominantly in the supine position (Fig. 1). The baseline characteristics of the study population and the balance between groups before and after IPW are described in Table 1. (More detailed data are shown in Additional file 1: Table E1.) Even though there were differences between groups (e.g., age, sex APACHE II score, comorbidities, previous days with oxygen therapy, and C-reactive protein), after weighting by IPW, the values of all variables were balanced, showing a standardized difference of less than 0.1 (Table 1 and Additional file 1: Fig. E1-E2). The variables related to oxygen therapy and exposure to prone positioning in the study groups are listed in Table 2 and Table E2 (Additional file 1). No patient received noninvasive ventilation with positive-pressure as respiratory support.

**Table 1:** Baseline characteristics of the study population before and after inverse probability of treatment weighting

Characteristic	Before IPW			After IPW^c^		
	Awake-prone positioning (*n* = 187)	Non-prone positioning (*n* = 148)	Standardized difference	Awake-prone positioning (*n* = 186)	Non-prone positioning (*n* = 147)	Standardized difference
Demographics						
Age, years, median (p 25–75)	57 (47–66)	66.5 (56.5–75)	− 0.58	60 (49–68)	62 (53–75)	− 0.07
Female, sex, *n* (%)	45 (24)	41 (28)	− 0.09	43 (23)	37 (25)	− 0.03
Body mass index	30 (27.5–33)	30.0 (26.8–33.1)	− 0.04	30.3 (28.4–34)	30 (27–33)	0.04
Comorbidities, *n* (%)						
Respiratory	22 (12)	22 (15)	− 0.07	24 (13)	19 (13)	0.02
Cardiovascular	41 (22)	43 (29)	− 0.15	48 (26)	44 (30)	− 0.03
Renal	8 (4,2)	9 (6)	− 0.10	14 (7.7)	8 (5.6)	− 0.03
Hepatic	4 (2)	1 (0.6)	0.12	3 (1,6)	3 (2)	− 0.06
Oncohematologic	2 (1)	13 (9)	− 0.38	6 (3.5)	9 (6)	− 0.05
Solid neoplasm	2 (1)	4 (2.7)	− 0.12	2 (1)	2 (1.35)	− 0.04
Immunosuppression	6 (3)	10 (6.7)	− 0.13	10 (5.4)	5 (3.3)	− 0.03
Neurologic	6 (3)	8 (5.4)	− 0.10	5 (2.5)	4 (2.9)	− 0.04
Diabetes	40 (21)	43 (29)	− 0.19	55 (29.5)	41 (28)	− 0.04
Hypertension	62 (33)	80 (54)	− 0.43	70 (37.2)	73 (50)	− 0.07
Smoking	50 (27)	53 (36)	− 0.20	51 (27.3)	41 (28)	− 0.01
Chronology						
Days from symptom onset to hospital admission, median (p25–75)	8 (6–10)	8.0 (5–10)	0.01	8 (6–10)	9 (5–11)	− 0.05
Previous days of oxygen therapy, median (p25–75)	1 (0–2)	1 (1–3)	− 0.26	1 (0–3)	1 (0–3)	0.003
Laboratory at admission						
CRP, mg/dl, median (p 25–75)	10.7 (5.1–18)	9.26 (5–17)	− 0.05	9.7 (5–16)	8.6 (5–17)	− 0.002
Respiratory-hemodynamics, and scores at admission						
APACHE score, median (p 25–75)	10 (7–12)	10 (8–13)	− 0.22	10 (6–13)	10 (7–13)	− 0.10
SOFA score, median (p 25–75)	3 (2–4)	3 (2–4)	0.02	3 (2–4)	3 (2–4)	− 0.05
CT score^d^, median (p 25–75)	13 (9–17)	13 (10–15)	0.12	13 (9–17)	13 (10–15)	0.01
ROX index, median (p 25–75)	6.6 (4.7–11)	6 (4.1–8.3)	0.39	6.3 (4.8–9.5)	5.6 (4.2–8.3)	0.10
Treatments						
Antibiotics, *n* (%)	150 (80)	133 (90)	− 0.28	158 (85)	128 (87)	− 0.07
Systemic corticosteroids, *n* (%)	185 (99)	145 (98)	0.08	184 (99)	146 (99)	0.03
Light sedation, *n* (%)	116 (62)	46 (31)	0.65	90 (48)	66 (45)	0.03
Oxygen therapy previous to ICU-admission (mode)^b^	1.68	1.71	− 0.07	1.78	1.75	− 0.02

The total number of awake-prone positioning and non-prone positioning patients is slightly different in the post-IPW pseudo-data set as a result of weighting

CRP: C-reactive protein; CT score: computed tomography score; HFNO: high-flow nasal oxygen; LDH: lactate dehydrogenase; PaCO_2_: CO_2_ blood pressure; PaO_2_/FiO_2_: ratio of pressure of oxygen in arterial blood (PaO_2_) to the fraction of inspired oxygen (FiO_2_). SaO_2_/FiO_2_: ratio of arterial oxygen saturation to fraction of inspired oxygen

^a^Numerical variables are expressed as median and percentile 25.75. Dichotomous categorical variables are expressed as *n* (%). The standardized difference > 0.1 represents an imbalance between the groups

^b^Before HFNO, patients received O_2_ in some of the three following ways: low-flow O_2_ cannula, Venturi mask, and non-rebreathing mask (see also Table 2). The value represents the mean distribution of the variable between groups

^c^Variables included in the logistic regression model with awake prone position as the main exposure

^d^Fifty-four patients did not have CT scans at the time of ICU admission. The values were imputed as the mean of each group population

**Table 2:** Treatment with oxygen therapy and prone positioning (see also, Additional file 1: Table E2)

Oxygen therapy and prone positioning	Awake prone positioning (*n* = 187)	Non-prone positioning (*n* = 148)
Previous to ICU-admission O_2_ therapy, *n* (%)		
Low-flow O_2_ Cannula	69 (37)	47 (32)
Venturi mask	8 (4)	5 (3)
Non-rebreathing mask	110 (59)	96 (65)
HFNO therapy and body position (at ICU)		
Days on HFNO, median (p 25–75)	5 (3–7)	3 (1.5–7)
Time of AW-PP (h/day), median (p 25–75)	12 (9–16)	0 (0–2)
Time on AW-PP (days), median (p 25–75)	5 (3–8)	0 (0–1)
Basal setting of HFNO		
Flow (liters/min), median (p 25–75)	60 (60–60)	60 (60–60)
FiO_2_, mean (SD)	0.6 (0.5–0.7)	0.6 (0.5–0.75)
HFNO at weaning		
FIow (liters/min), median (p 25–75)	40 (40–40)	40 (40–40)
FiO_2_, median (p 25–75)	0.3 (0.3–0.4)	0.3 (0.3–0.4)

AW-PP: awake prone position; FiO_2_: fraction of inspired oxygen; HFNO: O_2_ therapy with high-flow nasal cannula; ICU: intensive care unit

**Fig. 2:**
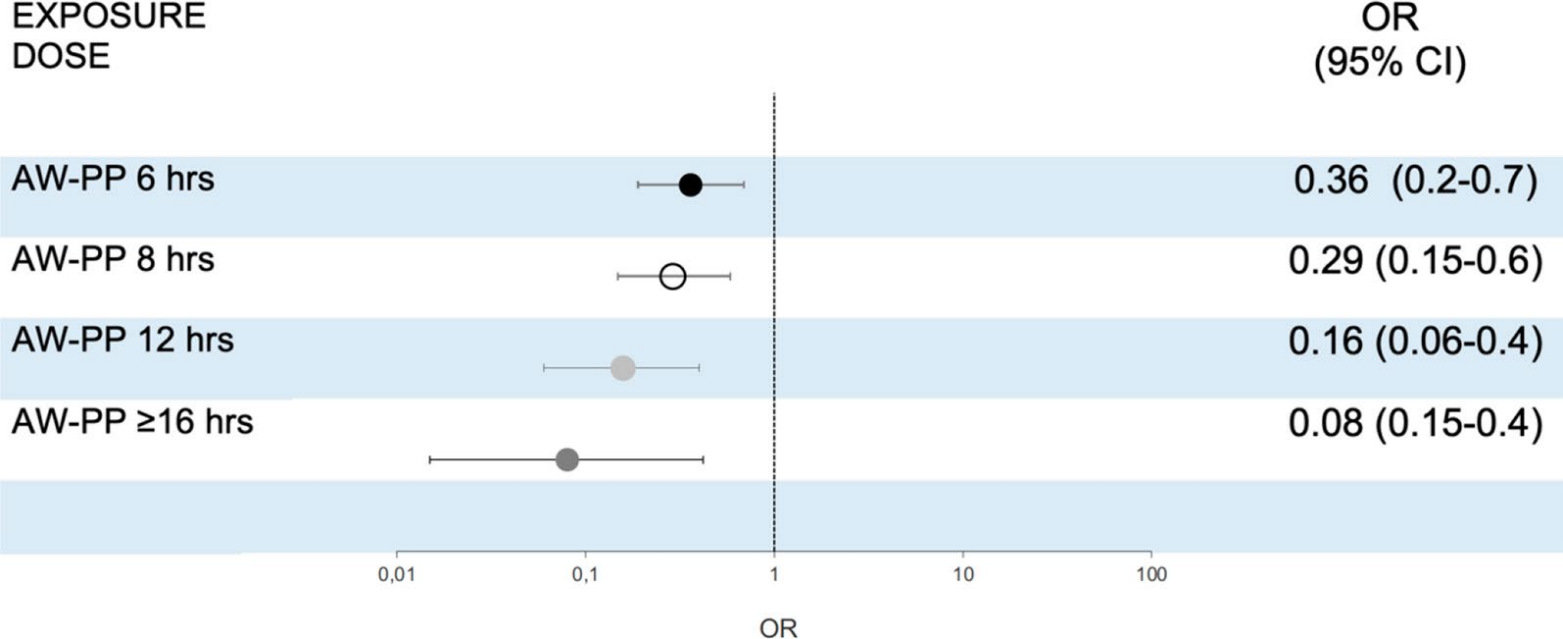
Risk of intubation between groups in awake prone position and non-prone position. OR indicates odds ratio; 95% CI indicates confidence interval. Non-awake prone positioning group (supine or lateral) as reference. Abbreviations. AW-PP: awake prone positioning. Weighted population and adjusted by center and pandemic time

Forty-four patients in the AW-PP group (23%) and 79 (53%) in NON-PP group were intubated (Additional file 1: Table E3). In the weighted population, the OR for endotracheal intubation was 0.27 (95% CI 0.14–0.47) and the adjusted OR by center and pandemic time in the weighted population was 0.36 (0.2–0.7) (Fig. 2 and Additional file 1: Table E4). The *e* value for the primary analysis for the effects of AW-PP on ETI was 2.72, showing robustness to potential unmeasured confounding (Additional file 1: Fig. E4). The reasons for ETI in the 123 patients who required it were progression of respiratory failure [*n* = 120 (98%)] and hemodynamic failure [*n* = 3 (2%)]. In the sensitivity analysis for ETI, the adjusted OR decreased progressively with increasing exposure to the prone position (measured in h/day), resulting in a dose–response effect (Fig. 2 and Additional file 1: Table E4). The effects of exposure to AW-PP on ETI were maintained when they were evaluated according to the severity of respiratory failure defined by the PaO_2_/FiO_2_ ratio (Additional file 1: Fig. E5). When the populations were compared based on the predominant position they adopted during the study period, differences were found between the AW-PP versus supine-position groups, but not between the AW-PP versus the lateral-position groups (Additional file 1: Fig. E6). The results of the analysis restricted to patients with AW-PP > 6 h versus patients with “zero” hour in prone position (*n* = 103) showed an OR = 0.29 (0.3–0.6) (Additional file 1: Fig. E7).

**Fig. 3:**
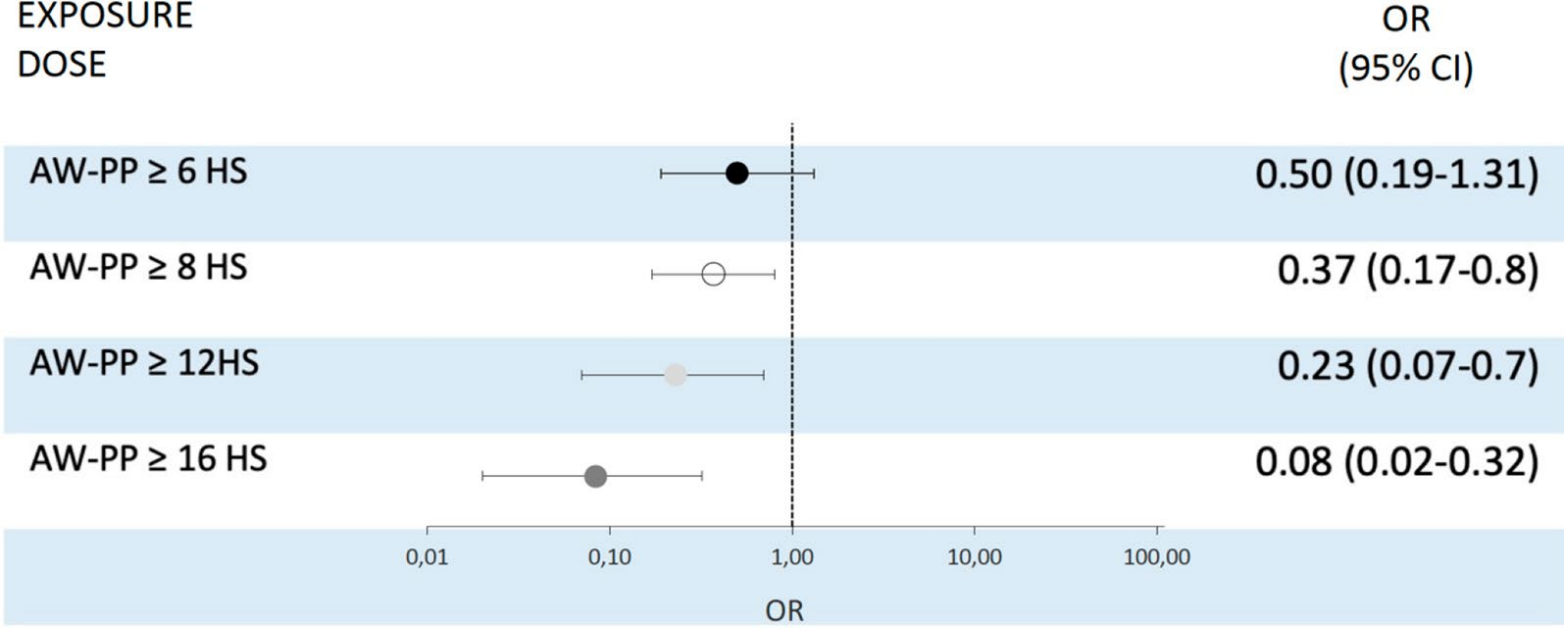
Risk of hospital mortality between groups in the awake prone position versus non- prone position. OR indicates odds ratio; 95% CI indicates confidence interval. Non-prone positioning group (supine or lateral) as reference. Abbreviations. AW-PP: awake prone positioning. Weighted population and adjusted by center and pandemic time

Twenty-one patients in the AW-PP group (11%) and 47 (32%) in the NON-PP group died while being hospitalized. In the weighted population, the OR for hospital mortality was 0.58 (0.19–1.77) and the adjusted OR by center and pandemic time in the weighted population was 0.50 (95% CI 0.19–1.31) (Fig. 3 and Additional file 1: Table E5). One death occurred in each group while patients remained on HFNO (prior to ETI). In the sensitivity analysis for hospital mortality, the adjusted OR progressively decreased with increasing exposure to the prone position (measured in h/day), with a true OR reduction when the exposure was ≥ 8 h/d [OR 0.37 (95% CI 95 0.17–0.8)] (Fig. 3 and Additional file 1: Table E5). The results of the analysis restricted to patients with AW-PP > 6 h versus patients with “zero” hours in prone position (n = 103) showed an OR = 0.37 (0.2–0.8) (Additional file 1: Fig. E7).

Other clinical outcomes are shown in Table E3 (Additional file 1). ICU and hospital length of stay for AW-PP and NON-AW-PP were [median (p 25–75)] 9 (6–14) vs. 12 (7–20) days (*p* = 0.0012); and 15 (11–25) vs. 20 (13–32) days, (*p* = 0.002), respectively. The time until endotracheal intubation for AW-PP and NON-PP was 3 (1–4) 1 versus 1 (1–3) 3 days (*p* = 0.017), respectively (Additional file 1: Fig. E7). The functional results are listed in Table E6 (Additional file 1), 95% of the patients who remained prone for at least 6 h managed to ambulate upon discharge from the ICU. The variables related to the respiratory parameters of ventilated patients are listed in Table E7 (Additional file 1). Three patients had severe complications in the AW-PP group: syncope in the prone position requiring ETI, cardiorespiratory arrest requiring ETI, and arterial line displacement with bleeding and hypotension (not requiring ETI), respectively.

## Discussion

In this multicenter prospective observational study of patients with COVID-19-related acute respiratory failure receiving initial treatment with HFNO, prone position for at least 6 h a day was associated with a lower risk of endotracheal intubation, even after adjustment for potential confounders. This association was more consistent as the exposure to prone position (measured in h/day) increased, thus evidencing a dose–response effect. Additionally, prone positioning was associated with a lower risk of hospital mortality when the exposure was > 8 h.

Since the beginning of the pandemic, decisions surrounding the proper time for initiation of invasive mechanical ventilation have been a matter of controversy [25, 26]. It has been suggested that delaying intubation could lead to patient self-induced lung injury. For this reason, some authors have recommended an early intubation strategy [27, 28]. Nonetheless, the potential benefits of such a strategy must be weighed against the complications associated with invasive mechanical ventilation, including higher risk of severe infections and death [6, 29]. Mortality in patients with COVID-19 receiving invasive mechanical ventilation is particularly high, with rates exceeding 70% in patients above 60 years [5], accentuating the need for caution when deciding which patients will benefit from this strategy.

Both prone position and HFNO may exert positive physiologic effects and improve outcomes. Prone position has been shown to improve ventilation–perfusion ratio [30], which may explain improved oxygenation, but also to decrease global lung strain and determine a more homogeneous distribution of regional strain [31, 32], which may prevent ventilation-induced lung injury. The prone position has been shown to reduce mortality in ventilated patients with moderate to severe ARDS when the exposure time is early and prolonged [9]. It is therefore recommended to use the prone position for a period > 12 h in these patients [33]. HFNO increases end-expiratory lung volume, decreases breathing effort and favors a more homogeneous ventilation distribution [34]. Besides, HFNO can reduce the rate of ETI in patients with acute hypoxemic respiratory failure and a PaO_2_/FiO_2_ ratio < 200 mmHg.[7].

The feasibility of applying HFNO and ventilation in AW-PP, on an independent or combined basis, has been evaluated in case series [11–13]. In our study, these interventions were applied concomitantly and systematically in a cohort of consecutive patients. Since study groups displayed differences in baseline variables, a causal approximation was carried out by weighting these variables using a propensity score method (IPW). The results displayed a balanced distribution of values of the variables between groups, thereby allowing the assumption of population interchangeability [18]. In view of the fact that the attending healthcare teams may have acted differently as they gained experience over the course of the pandemic [35], a second adjustment was made based on the time of the pandemic when the patients were included and the centers (“double robust”). The same method was used in the sensitivity analysis for restricted groups. The results obtained from the secondary outcome provide additional consistency to the results of the primary analysis.

A recent meta-trial comparing HFNO versus HFNO plus AW-PP showed a reduction in ETI risk in the combined treatment group. Importantly, the size of the effect was small and there were no benefits on mortality or other relevant clinical outcomes [14]. These results can be explained by the short exposure time to AW-PP in the treatment group (5 h). The rate of treatment failure (mainly ETI) was lower in patients who remained on AW-PP > 8 h/d. However, the study was not designed to evaluate the effect of exposure time in AW-PP, and no comparisons were made between intervention and control groups according to exposure time [14].

Different physiological parameters improved from the beginning of the prone position and progressively increased with exposure time within each session, up to 16–24 h in ventilated patients [36, 37]. On the other hand, evidence from observational studies suggests that a period > 24 h/session may not add additional clinical benefits [38, 39]. The rate of adherence in our study was high. Fifty-six percent of patients met the definition of AW-PP (≥ 6 h/d), placed in the prone position for a large number of hours (12 h/d), thereby resulting in a greater reduction in ETI risk. That exposure time could explain the differences in the effect size (risk of ETI) in our study, with respect to the meta-trial. Additionally, our results suggest that ≥ 8 h/d may be the time necessary to impact on death risk reduction.

Our study has several strengths: (a) a representative sample of patients with COVID-19-related ARF from different ICUs with consecutive inclusion, and similar initial treatment (HFNO), thus minimizing selection bias; (b) exhaustive treatment of confounders using causal inference and a sensitivity analysis that showed consistent results in different sub-populations. The main limitations of this study are those inherent to establishing causality by an observational design, i.e., the possibility of not considering unmeasured confounders. Several strategies were employed to minimize the potential biases inherent to the design: The prospective nature of the cohort allowed us to consider most of the known confounders; the use of IPW in the adjustment permitted to reduce multidimensionality and balance the factors that could influence the hypothesis [23], and the estimation of the *e* value [24]. All the patients included were treated in an ICU setting. Therefore, these results cannot be extrapolated to patients seen in a less complex settings (a general ward), or with less severe diseases. Finally, another limitation was the potential bias derived from the non-blinded position of the attending team to intubation order. Nonetheless, the healthcare team followed the recommendations pre-established in the protocol.

## Conclusions

The results of our study indicate that, in patients with Covid-19 and acute respiratory failure admitted to the ICU and initially treated with HFNO, prone positioning for at least 6 h/day reduces the risk of endotracheal intubation, and for ≥ 8 h/d the risk of death.

## Supplementary Information

**Additional file 1:** 1. Further details on the procedures. 2. Further details on statistical analysis. **TABLE E1.** Baseline characteristics of the study population and the balance between groups. **TABLE E2.** Treatment with oxygen therapy and prone positioning. **TABLE E3.** Outcomes of patients in awake prone positioning versus non awake prone positioning. **TABLE E4.** Risk of intubation in awake prone positioning versus non awake prone positioning. OR indicates odds ratio; 95% CI indicates confidence interval. Non-prone positioning group as reference. **TABLE E5.** Risk of hospital mortality in awake prone positioning versus non-prone positioning. **TABLE E6.** Functional outcomes at discharge. **TABLE E7.** Variables related to invasive mechanical ventilation in ventilated patients at day 1 after starting invasive mechanical ventilation. **TABLE E8.** Selection of variables for adjustment of confounding. **Figure E1.** Diagnosis of inverse probability weights-propensity score (graphic and statistical). **Figure E2.** Standardized differences before and after applying inverse probability weighting.** Figure E3.** Directed acyclic graph (DAG). **Figure E4.** E-value calculation for primary outcome of interest (ETI). **Figure E5.** Risk of intubation between groups in the awake prone position vs. non-awake prone position according to severity of respiratory failure. **Figure E6.** Risk of intubation between groups in the awake prone position vs. non-awake prone position according to predominant body position. **Figure E7.** Cumulative incidence of endotracheal intubation over time in the study population.

## Data Availability

The datasets used and/or analyzed during the current study are available from the corresponding author on reasonable request.

## Abbreviations

ARDS: Acute respiratory failure and acute respiratory distress syndrome
ARF: Acute respiratory failure
AW-PP: Awake prone positioning
CI: Confidence intervals
CRP: C-reactive protein
CT score: Chest computed tomography score
DAG: Direct acyclic graph
ETI: Endotracheal intubation
H/DAY: Hours per day
HFNO: High-flow nasal oxygen
ICU: Intensive care unit
IPW-PS: Inverse probability weighting–propensity score
NON-PP: Non-prone positioning
OR: Odds ratio
ROX index: Respiratory rate oxygenation index
SpO_2_: Peripheral oxygen saturation
STROBE: Strengthening the reporting of observational studies in epidemiology
