# Supplementary material for: Impact of exposure time in awake prone positioning on clinical outcomes of patients with COVID-19-related acute respiratory failure treated with high-flow nasal oxygen: a multicenter cohort study
Source: Crit Care. 2022 Jan 7;26:16. doi: 10.1186/s13054-021-03881-2 (PMC8740872; doi:10.1186/s13054-021-03881-2)
Supplement: Supplementary file 1 — Additional file 1: 1. Further details on the procedures. 2. Further details on statistical analysis. TABLE E1. Baseline characteristics of the study population and the balance between groups. TABLE E2. Treatment with oxygen therapy and prone positioning. TABLE E3. Outcomes of patients in awake prone positioning versus non awake prone positioning. TABLE E4. Risk of intubation in awake prone positioning versus non awake prone positioning. OR indicates odds ratio; 95% CI indicates confidence interval. Non-prone positioning group as reference. TABLE E5. Risk of hospital mortality in awake prone positioning versus non-prone positioning. TABLE E6. Functional outcomes at discharge. TABLE E7. Variables related to invasive mechanical ventilation in ventilated patients at day 1 after starting invasive mechanical ventilation. TABLE E8. Selection of variables for adjustment of confounding. Figure E1. Diagnosis of inverse probability weights-propensity score (graphic and statistical). Figure E2. Standardized differences before and after applying inverse probability weighting. Figure E3. Directed acyclic graph (DAG). Figure E4. E-value calculation for primary outcome of interest (ETI). Figure E5. Risk of intubation between groups in the awake prone position vs. non-awake prone position according to severity of respiratory failure. Figure E6. Risk of intubation between groups in the awake prone position vs. non-awake prone position according to predominant body position. Figure E7. Cumulative incidence of endotracheal intubation over time in the study population. [file 13054_2021_3881_MOESM1_ESM.docx]

**Online Supplement**

**Impact of awake prone positioning in clinical outcomes of patients with COVID-19 related acute respiratory failure treated with high-flow nasal oxygen: a multicenter cohort study**

**Argentine Collaborative Group on High Flow and Prone Positioning**

**Participating centers**

Hospital Privado de Comunidad. Mar del Plata. Argentina

Clínica Olivos, SMG. Olivos. Buenos Aires. Argentina

Hospital Universitario Austral. Pilar. Buenos Aires. Argentina

Sanatorio Clínica Modelo de Morón. Morón. Buenos Aires. Argentina

Hospital Alemán. Ciudad Autónoma de Buenos Aires. Argentina

Sanatorio de la Trinidad Palermo. Ciudad Autónoma de Buenos Aires. Argentina

**University Affiliated Research Center**

Hospital Privado de Comunidad: Universidad de Buenos Aires. Buenos Aires, Argentina.

Hospital Alemán: Universidad del Salvador. Buenos Aires, Argentina.

Hospital Universitario Austral: Universidad Austral, Pilar, Buenos Aires, Argentina.

Sanatorio Clínica Modelo de Morón: Universidad de Morón, Buenos Aires, Argentina

**Supplementary Appendix**

#

[**FURTHER DETAILS ON THE PROCEDURES** 2](#_heading=h.3dy6vkm)

[**FURTHER DETAILS ON STATISTICAL ANALYSIS** 3](#_heading=h.1t3h5sf)

[Inverse probability weighting-propensity score 3](#_heading=h.4d34og8)

[Selection of variables for adjustment of confounding 3](#_heading=h.2s8eyo1)

[**TABLES AND FIGURES** 5](#_heading=h.17dp8vu)

[TABLE E1: Baseline characteristics of the study population and the balance between groups. 5](#_heading=h.3rdcrjn)

[TABLE E2- Treatment with oxygen therapy and prone positioning 7](#_heading=h.lnxbz9)

[TABLE E3: Outcomes of patients in awake prone positioning versus non awake prone positioning. 8](#_heading=h.44sinio)

[TABLE E4: Risk of intubation in awake prone positioning versus non awake prone positioning. OR indicates odds ratio; 95% CI indicates confidence interval. Non-prone positioning group as reference. 8](#_heading=h.2jxsxqh)

[TABLE E5: Risk of hospital mortality in awake prone positioning versus non-prone positioning. 8](#_heading=h.3j2qqm3)

[TABLE E6: Functional outcomes at discharge 9](#_heading=h.1y810tw)

[TABLE E7: Variables related to invasive mechanical ventilation in ventilated patients at day 1 after starting invasive mechanical ventilation start 9](#_heading=h.4i7ojhp)

[TABLE E8- Selection of variables for adjustment of confounding 10](#_heading=h.1ci93xb)

[Figure E1. Diagnosis of inverse probability weights-propensity score (graphic and statistical) 11](#_heading=h.3whwml4)

[Figure E2. Standardized differences before and after applying inverse probability weighting. 12](#_heading=h.2bn6wsx)

[Figure E3: Directed acyclic graph (DAG) 13](#_heading=h.qsh70q)

[Figure E4. E-value calculation for primary outcome of interest (ETI) 14](#_heading=h.3as4poj)

[Figure E5: Risk of intubation between groups in the awake prone position vs. non-awake prone position according to severity of respiratory failure. 15](#_heading=h.1pxezwc)

[Figure E6: Risk of intubation between groups in the awake prone position vs. non-awake prone position according to predominant body position. 16](#_heading=h.49x2ik5)

[Figure E7. Cumulative incidence of endotracheal intubation over time in the study population 17](#_heading=h.2p2csry)

[**REFERENCES** 18](#_heading=h.3o7alnk)

#

# **FURTHER DETAILS ON THE PROCEDURES**

Patients were admitted to the ICU either from the general ward or the emergency room. Immediately after admission to the ICU, the criteria about oxygenation and/or work of breathing were confirmed. Then, a high-flow nasal cannula sized according to the nares size (Optiflow, Fisher and Paykel Healthcare) was placed, and connected to a specific device for the provision of high-flow O_2_ (Humidoflo HF-2900, GGM Co., Taiwan; or AIRVO, Fisher and Paykel Healthcare, Auckland, New Zealand) or to an ICU respirator in high flow mode (Neumovent GraphNet Advance, Tecme S.A., Córdoba, Argentina; or Monnal T75, Air Liquide Medical System, France). Once therapy with HFNO was started, participants were encouraged and assisted by the health-care team to rotate from supine to prone position for as long as possible, taking breaks for personal hygiene and eating. No maximum time limits for prone position were established. The patients were in the Intensive Care Unit during the entire study period; and, therefore, remained under the direct care of the health team. At the end of each 8-hour shift, nurses and physiotherapists reported the number of hours in the prone position in the medical records. When they had doubts as to the precise time elapsed, the patients were asked to estimate the time of exposure.

Where prone positioning was not tolerated by patients, they were assisted to remain in the lateral position, alternating right and left decubitus for as long as they could bear it. Supine position was allowed where patients could not tolerate any of the positions above mentioned. These interventions were maintained during the study period until one of the following criteria was met: maintenance of SpO_2_ > 92% with FiO_2_ ≤ 40%, and flow ≤ 40 L/min for a period > 12 hours in the supine position; or endotracheal intubation.

All centers have over 5 years of experience in non-invasive mechanical ventilation approach for critically ill patients. Participating ICUs comprised intensive care physicians, respiratory therapists, and critical care nurses.

The initial flow was 50-70 L/min, with the FiO_2_ necessary to obtain an SO2 > 92%. No maximum FiO_2_ limits were established. Once clinical and gas stability were attained, flow and oxygen was progressively weaned. The de-escalation was initiated with a gradual FiO_2_ decrease until ≤40% was reached. Subsequently, the flow level was progressively reduced until reaching ≤40 L/min. Once these requirements were met, a low-flow nasal cannula was placed with an O_2_ flow of 3-5 L/min in the supine position. Analgesic drugs (opioids, paracetamol) or light sedation (dexmedetomidine) was allowed and indicated according to the criteria of the health-care team.

The following data was recorded: a) the predominant position adopted by the patient, defined as the position in which the patient spent most h/day, i.e., prone, lateral or supine position; b) the average number of h/day in that position; and c) the number of days of exposure to said position for a period of ≥ 6 h/day (AW-PP).

# **FURTHER DETAILS ON STATISTICAL ANALYSIS**

## Inverse probability weighting-propensity score

Since treatment allocation was not randomly assigned, inverse probability of treatment weighting (IPW) was used to control for potential confounding by indication. The IPW is an extension of the propensity score method used to summarize the conditional probability of treatment assignment [[1,2]](https://paperpile.com/c/Z8qVnR/9PM1+Czmp). Using the IPW method, the weights are the inverse probability of assigning the treatments derived from the logistic model with treatment as the dependent variable and all observed confounders as the independent variables. The adjusted relationship between treatment and outcome can then be estimated using inverse probability-weighted regression models where treatment is the sole independent variable. The theoretical advantage of this method is that it allows to adjust for more confounders, requiring fewer distributional assumptions of the underlying data, and having the capacity to incorporate time-dependent covariates and deal with censored data, as compared to other propensity score methods [[2]](https://paperpile.com/c/Z8qVnR/Czmp). We evaluated and reported the balance diagnosis of IPW according to Austin and Stuart [[3,4]](https://paperpile.com/c/Z8qVnR/Zp6j+BMlU).

To calculate the inverse probability of treatment weights, the patient’s propensity to undergo AW-PP was estimated, using a logistic-regression model that included predictor variables selected on the basis of their *a priori* possibility of confounding the relationship between body positioning and endotracheal intubation (age, sex, BMI, comorbidities, smoking status, SOFA score, APACHE II score, days from symptoms onset to hospital admission, previous use of O_2_ (days and administration mode), ROX index, CT score and CRP at admission) (Table E-1). Those patients who had undergone an AW-PP were assigned a weight of 1/(propensity score) and those who had undergone a non AW-PP, a weight of 1/(1 − propensity score). To reduce variability in the inverse probability of treatment–propensity score, stabilized weights were applied. The 1% and 99% percentiles were used to eliminate those observation units with extreme IPWT values [[5]](https://paperpile.com/c/Z8qVnR/8apS0) . To check the covariables balance in the pseudo population, we used graphic and statistical methods (See Figure E1 and Figure E2). Measures of association are expressed as odds ratios (OR) with 95% confidence intervals (CI), and which were created using bootstrapping to account for correlation of the weighted population.

## Selection of variables for adjustment of confounding

Covariate adjustment poses the risk of inducing selection bias if conditioning is performed on variables that act as colliders. The structural relationship between the exposure of interest and outcome, including all other potential covariates can be depicted using a direct acyclic graph (DAG) [[6–8]](https://paperpile.com/c/Z8qVnR/UvoPp+EKfL5+zS7pN). This form of causal diagram is constructed from subject matter knowledge including previous research, and describes the roadmap of causal connections between variables of interest. By assessing the relations between variables, DAGs help to identify potential sources of bias and how to control them when estimating causal associations. The DAG used in this study is shown in Figure E3; and was constructed with DAGitty [[9]](https://paperpile.com/c/Z8qVnR/NQXL7). Using DAG, we identified five nodes as controlled PS-IPW; namely the severity of respiratory failure and variables related to COVID-19, the baseline variables, therapies and variables related to prone position. Additionally, the participating center and chronological time since the onset of the pandemic were included as independent variables in a logistic regression model to adjust for the potential confusion effect (doubly robust approach). For all nodes, we identified the variables that could be used to control for confounding, Table 1 shows each node and the selected variables.

Estimation of E-value

Unmeasured confounding represents a major concern when estimating causal effects in observational studies. Thus a metrics that calculates how strong an unmeasured confounder should be in order to overturn the reported results is advisable. The E-value, which is presented in the risk ratio scale (with values ranging from 1 to infinite), represents the strength of association that a confounder should present with both the exposure and the outcome to explain away the estimated findings [[10]](https://paperpile.com/c/Z8qVnR/um3gp). The E-value was calculated for the primary outcome using an E-value calculator: <https://www.evalue-calculator.com>

# **TABLES AND FIGURES**

## **TABLE E1: Baseline characteristics of the study population and the balance between groups.**

|  | **Awake prone positioning**  (n=187) | **Non-prone positioning**  (n=148) | **Standardized**  **difference** |
| --- | --- | --- | --- |
| Age, years | 57 (47-66) | 66.5 (56-75) | -0.62 |
| Female sex, n (%) | 45 (24) | 42 (28) | -0.28 |
| Body Mass Index | 30 (27-34) | 30 (27-33) | 0.08 |
| **Comorbidities, n (%)** | | | |
| Respiratory | 23 (14.8) | 22 (12.3) | -0.05 |
| Cardiovascular | 42 (22.4) | 43 (29) | -0.02 |
| Renal | 8 (4.3) | 10 (6.8) | -0.01 |
| Hepatic | 4 (2.1) | 1 (0.7) | 0 |
| Oncohematologic | 2 (1) | 14 (9.5) | -0.46 |
| Solid neoplasm | 2 (1) | 4 (3) | -0.18 |
| Immunosuppression | 7 (3.8) | 10 (6.8%) | -0.22 |
| Neurologic | 6 (3.2%) | 8 (5.4) | -0.37 |
| Diabetes | 39 (20.9) | 43 (29) | 0.03 |
| Hypertension | 62 (33.1) | 80 (54) | -0.40 |
| Smoking | 50 (26.7) | 53 (36) | -0.16 |
| **Chronology** | | | |
| Days from symptom onset to hospital admission | 5 (3-8) | 4 (2-7) | -0.08 |
| Days from symptom onset to HFNO | 8 (6-11) | 8 (5-10) | -0.25 |
| Previous days of oxygen therapy | 1 (0-2) | 1 (1-3) | -0.30 |
| **Laboratory at admission** | | | |
| LDH, IU/L | 380 (301-501) | 383 (289-523) | -0.13 |
| D-dimer, ng/ml | 749 (410-1338) | 667 (330-1107) | 0.17 |
| CRP, mg/dl | 10.7 (5-18) | 9.3 (5. -18) | 0.28 |
| Ferritin, ng/ml | 1512 (757-2607) | 1447 (666-2530) | -0.15 |
| **Respiratory-hemodynamics, scores at admission** | | | |
| APACHE score | 10 (7-12) | 10 (8-13) | -0.14 |
| SOFA score | 3 (2-4) | 3 (2-4) | 0.04 |
| CT score | 13 (9-17) | 13 (10-15) | 0.02 |
| PaO2/FiO2, mmHg | 130 (115-185) | 129 (97-176) | 0.14 |
| PaCO2, mmHg | 35 (33-38) | 35 (33-37) | 0.05 |
| SaO2/FiO2 | 158 (123-265) | 136 (118-230) | 0.30 |
| ROX index | 6.6 (4.9-10.9) | 6 (4.1-6.3) | 0.40 |
| Respiratory Rate | 26 (22-30) | 27 (24-30) | -0.21 |
| Use of accessory respiratory muscles, n(%) | 63 (34%) | 66 (44.6%) | -0.13 |
| Mean arterial pressure, mmHg. | 89 (82-100) | 89 (83-95) | 0.20 |
| Heart rate, beats/min | 82 (72-91) | 82 (75-89) | -0.13 |
| **Treatments, n (%)** | | | |
| Antibiotics | 151 (80.7) | 133 (90.5) | -0.15 |
| Systemic corticosteroids | 186 (99.5%) | 146 (99.3) | -0.16 |

Values are expressed as a median with their percentiles 25-75%, and the frequencies as numbers and percentages.

The standardized difference > 0.1 represents an imbalance between the groups.

Abbreviations. CRP: C-reactive protein; CT score: computed tomography score; HFNO: high flow nasal oxygen; LDH: lactate dehydrogenase; PaCO2: CO2 blood pressure; PaO2/FiO2: ratio of pressure of oxygen in arterial blood (PaO2) to the fraction of inspired oxygen (FiO2). SaO2/FiO2: ratio of arterial oxygen saturation to fraction of inspired oxygen.

##

## **TABLE E2- Treatment with oxygen therapy and prone positioning**

|  | **AW-PP**  **(n=187)** | | **Non-PP**  **(n=148)** | |
| --- | --- | --- | --- | --- |
|  |  |  | **Lateral**  **(n=84)** | **Supine**  **(n=64)** |
| Percentage of patients | 56 | | 25 | 19 |
| Time in AW- PP, h/day, median (p25-75)  mean (SD) | 12 (9.2-16)  12.5 (3.92) | | 0 (0-2)  1.06 (1.87) | 0 (0.2)  0.98 (1.4) |
| Time in AW-PP (days), median (p25-75)  mean (SD) | 5 (3-8)  5.8 (3.6) | | 0 (0-1)  0.85 (1.73) | 0 (0-2)  1.25 (1.7) |
| **BASAL SETTING OF HFNO** | | | | |
| FIow (lts/min), median (p25-75) | | 60 (60-60) | 60 (55-60) | 60 (57,5-60)) |
| FiO2, median (p25-75) | | 0.6 (0.5-0.7) | 0.6 (0.5-0.7) | 0.7 (0.5-0.8) |
| **WEANING SETTING OF HFNO** | | | | |
| FIow (lts/min), median (p25-75) | | 40 (40-40) | 40 (40-40) | 40 (35-40) |
| FiO2, median (p25-75) | | 0.3 (0.3-0.4) | 0.35 (0.3-0.5) | 0.35 (0.3-0.4) |

Abbreviations. AW-PP: awake prone position; FiO_2_: fraction of inspired oxygen; HFNO: O_2_ therapy with high flow nasal cannula; ICU: intensive care unit. Non-PP: Non-prone position

##

## **TABLE E3: Outcomes of patients in awake prone positioning versus non awake prone positioning.**

| **Outcome** | **Awake prone positioning**  **(n=187)** | **Non-prone positioning**  **(n=148)** | **p value*** |
| --- | --- | --- | --- |
| Endotracheal intubation, n (%) | 44 (23) | 79 (53) | <0.0001 |
| In-hospital mortality n (%) | 21 (11) | 47 (32) | <0.0001 |
| Mortality at ICU discharge, n (%) | 20 (11) | 47 (32) | <0.0001 |
| Mortality, Ventilated patients, n (%) | 19/44 (43) | 46/79 (58) | 0.086 |
| ICU LOS, days, median (p 25-75) | 9 (6-14) | 12 (7-20) | 0.0012 |
| Hospital LOS, days, median (p25-75) | 15 (11-25) | 20 (13-32) | 0.002 |
| VFDs (ventilated patients) | 12 (4-17) | 15 (8-20) | 0.15 |

Abbreviations. Hospital LOS: length of in-hospital stay. ICU: intensive care unit. ICU-LOS: Length of stay in ICU; VFDs: ventilator-free days, at day 28.

OR indicates odds ratio; 95% CI indicates confidence interval. Non awake prone positioning group as reference.

## **TABLE E4: Risk of intubation in awake prone positioning versus non awake prone positioning. OR indicates odds ratio; 95% CI indicates confidence interval. Non-prone positioning group as reference.**

| **Awake prone position** | **OR*** | **Adjusted OR¶** |
| --- | --- | --- |
| ≥ 6 hours | 0.27 (0.14-0.47) | 0.36 (0.2-0.7) |
| ≥ 8 hours | 0.22 (0.12-0.41) | 0.29 (0.15-0.6) |
| ≥ 12 hours | 0.12 (0.05-028) | 0.16 (0.06-0.4) |
| ≥16 hours | 0.05 (0.012-024) | 0.08 (0.015-0.4) |

OR indicates odds ratio; 95% CI indicates confidence interval. Non-prone positioning group as reference.

*Weighted population (by inverse probability weight)

**¶** Weighted population adjusted by center and pandemic time.

## **TABLE E5: Risk of hospital mortality in awake prone positioning versus non-prone positioning.**

| **Awake prone position** | **OR*** | **Adjusted OR¶** |
| --- | --- | --- |
| ≥ 6 hours | 0.58 (0.19-1.77) | 0.50 (0.19-1.31) |
| ≥ 8 hours | 0.43 (0.14-1.35) | 0.37 (0.17-0.8) |
| ≥ 12 hours | 0.15 (0.06-0.37) | 0.23 (0.07-0.7) |
| ≥16 hours | 0.31 (0.11-0.88) | 0.08 (0.02-0.32) |

OR indicates odds ratio; 95% CI indicates confidence interval. Non-prone positioning group as reference.

*Weighted population (by inverse probability weight)

**¶** Weighted population adjusted by center and pandemic time.

## **TABLE E6: Functional outcomes at discharge**

|  | **Awake prone positioning** | **Non-prone positioning** | **p** |
| --- | --- | --- | --- |
| ICU-acquired weakness (n=240), n (%) | 23/151 (15) | 27/89 (30) | 0.005 |
| Delirium (n=238), n (%) | 20/151 (13) | 28/87 (32) | <0.001 |
| Ambulation at hospital discharge  (n=242), n (%) | 142/159 (95) | 73/92 (79) | <0.001 |
| Pressure ulcer (n=242) | 9/152 (6) | 12/90 (13) | 0.048 |

Values are reported for surviving patients.

## **TABLE E7: Variables related to invasive mechanical ventilation in ventilated patients at day 1 after starting invasive mechanical ventilation start**

|  | **Awake prone positioning** | **Non-prone positioning** | **p** |
| --- | --- | --- | --- |
| PaO2/ FiO2, mmHg. | 202 (150-240) | 176 (128-264) | 0.62 |
| Tidal volume, ml. | 425 (300-490) | 425 (300-460) | 0.20 |
| Respiratory system compliance, ml/cm H_2_O | 41 (30-50) | 34 (30-41) | 0.15 |
| Driving pressure, cm H_2_O | 10 (10-12) | 11 (10-13) | 0.41 |
| PEEP, cm H_2_O | 12 (10-14) | 12 (10-12) | 0.58 |

Values are expressed as a median (p 25-75).

Abbreviations. PaO_2_/FiO_2_: ratio of pressure of oxygen in arterial blood (PaO_2_) to the fraction of inspired oxygen (FiO_2_).

##

## **TABLE E8- Selection of variables for adjustment of confounding**

| Node | Variables included in the PS-IPW | Variables not included in the PS -IPW |
| --- | --- | --- |
| Baseline variables | Comorbidities (Respiratory,  Cardiovascular, Renal, Hepatic, Oncohematologic, Solid neoplasm, Immunosuppression, Neurologic, Diabetes, Hypertension)  Age  Sex  BMI  Smoking status |  |
| Variables related to COVID-19 | Days from symptoms onset to hospital admission  Previous use of O_2_ (days and administration mode) |  |
| Severity of respiratory failure | SOFA  APACHE  ROX-I  TC Score  C-reactive protein | HR*  RR*^#^  Mean arterial  Pressure*  PaO_2_/FiO_2_*  PaCO_2_^&^  SaO_2_/FiO_2_^#^  Use of accessory respiratory muscles ^&^ |
| Therapies | Previous use of O_2_ (days and administration mode  Antibiotic therapy  Corticosteroid therapy |  |
| Variables related to prone position | Light sedation  Age  Sex  BMI |  |
| ICUs |  | Participating center |
| Time since the onset of the pandemic |  | Time since the onset of the pandemic ^Ç^ |

*variables included in severity scores.

#variables included in ROX index.

& variables with 13% missing data. It was decided to exclude said variables from the model and not the impute the data since they were represented by other variables included in the PS-IPW model.

Ç included as independent variables in the double robust approach adjustment.

Abbreviations. BMI: body mass index. HR: heart rate. PaO_2_/FiO_2_: ratio of pressure of oxygen in arterial blood (PaO2) to the fraction of inspired oxygen (FiO2). PS-IPW: propensity score-inverse probability weight. RR: respiratory rate. SpO2/FiO2: ratio of peripheral arterial oxygen saturation to the inspired fraction of oxygen

## **Figure E1. Diagnosis of inverse probability weights-propensity score (graphic and statistical)**


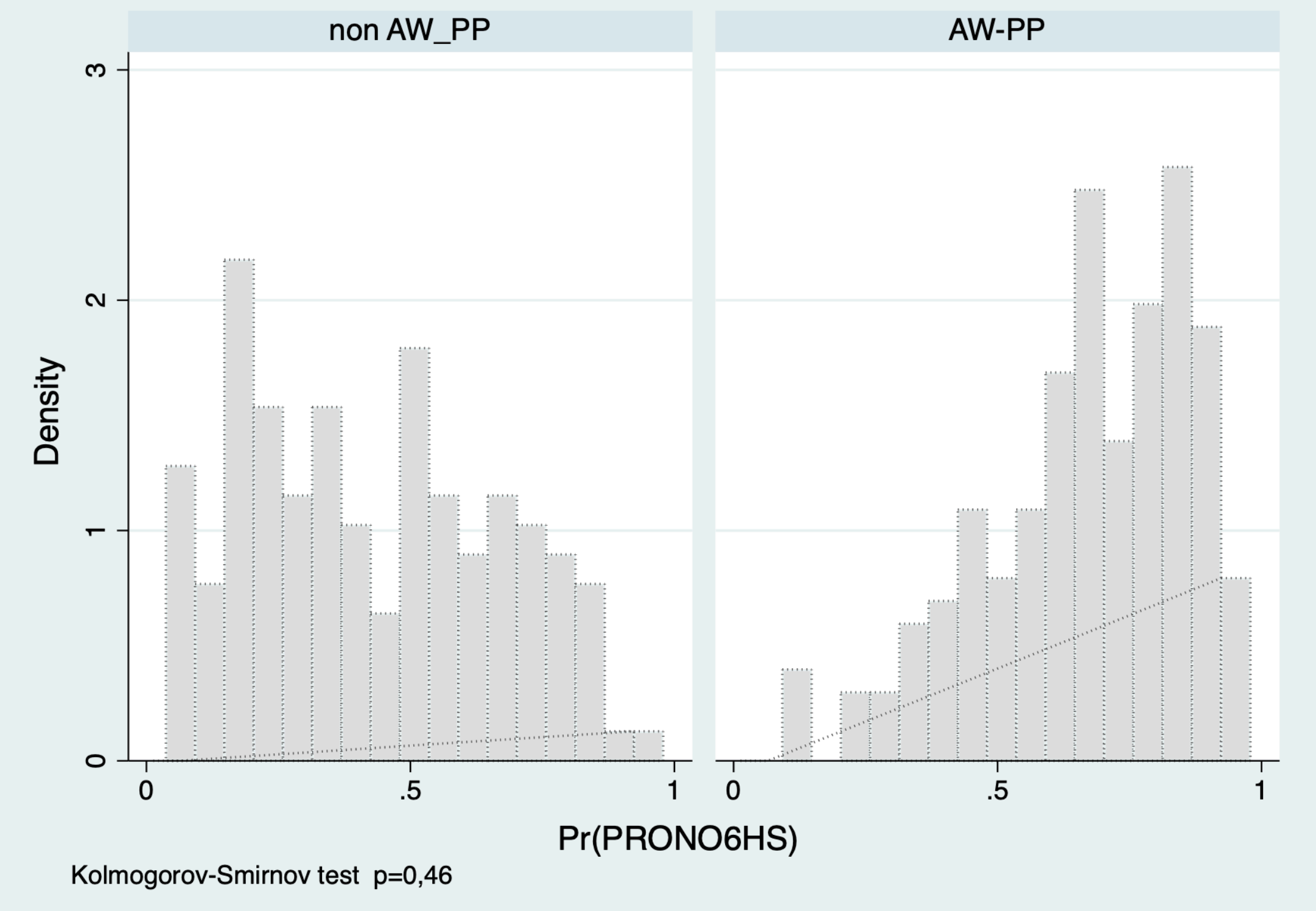


**NON-PP**

## **Figure E2. Standardized differences before and after applying inverse probability weighting.**


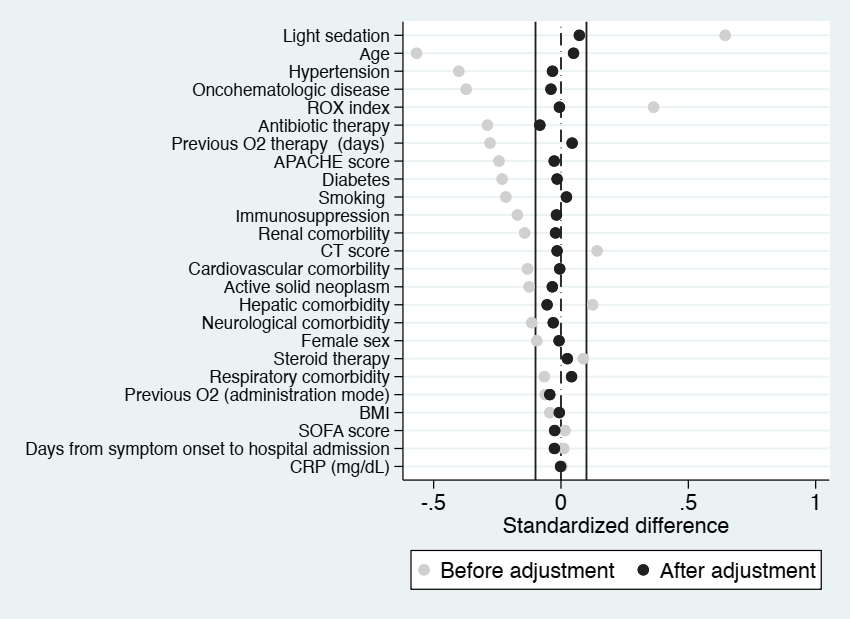


This figure shows the absolute standardized differences between AW-PP and non-PP patients for baseline covariates selected in the unweighted sample and after applying the weights derived from the inverse probability weighting. The vertical dashed line denotes a standardized difference of 0.1. Effect sizes below 0.1 are considered to be very small. This indicates that the baseline characteristics were well balanced between AW-PP and non-PP patients after applying the weighting.

Abbreviations. AW-PP: awake prone positioning

## **Figure E3: Directed acyclic graph (DAG)**


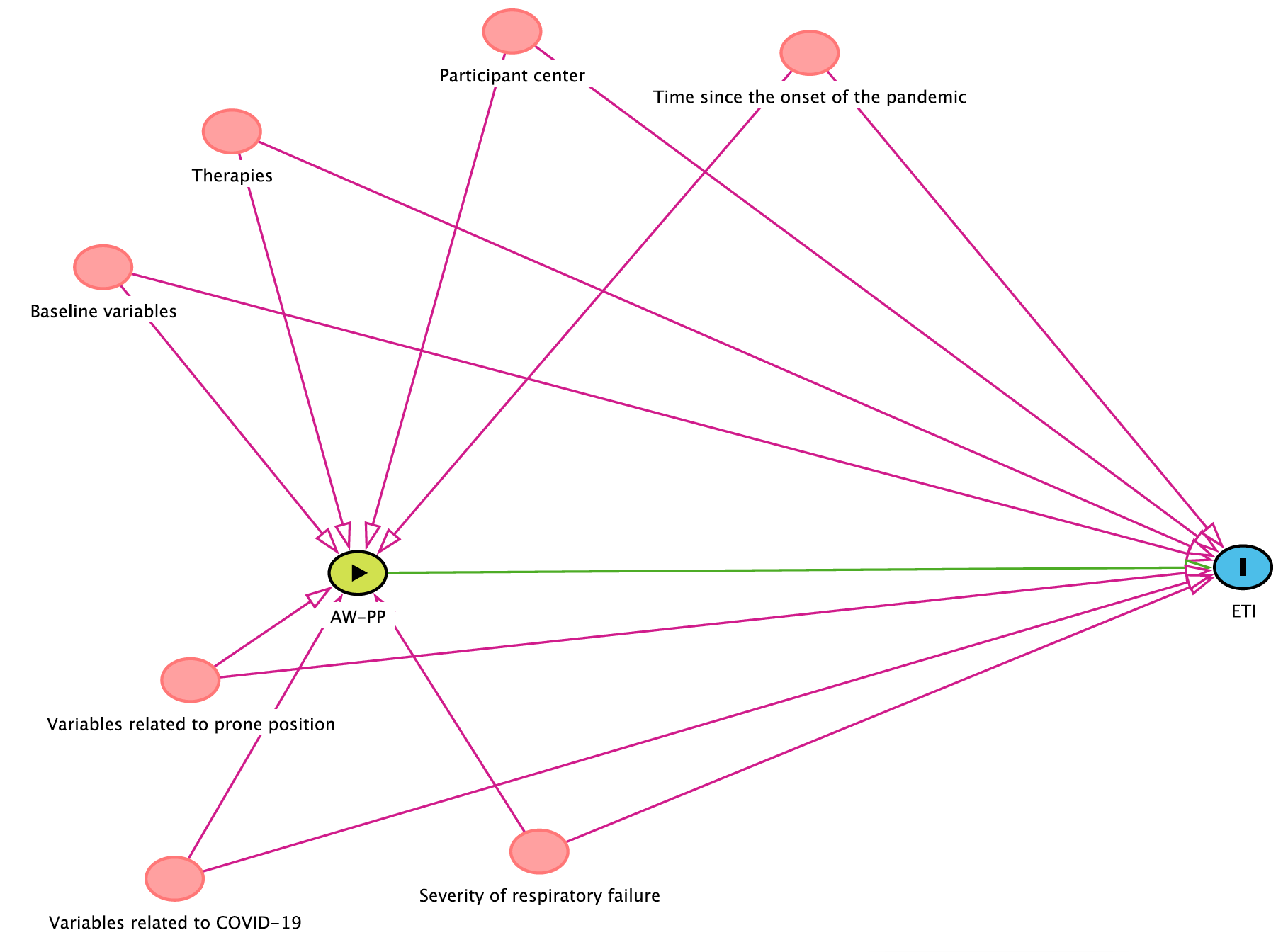


## **Figure E4. E-value calculation for primary outcome of interest (ETI)**


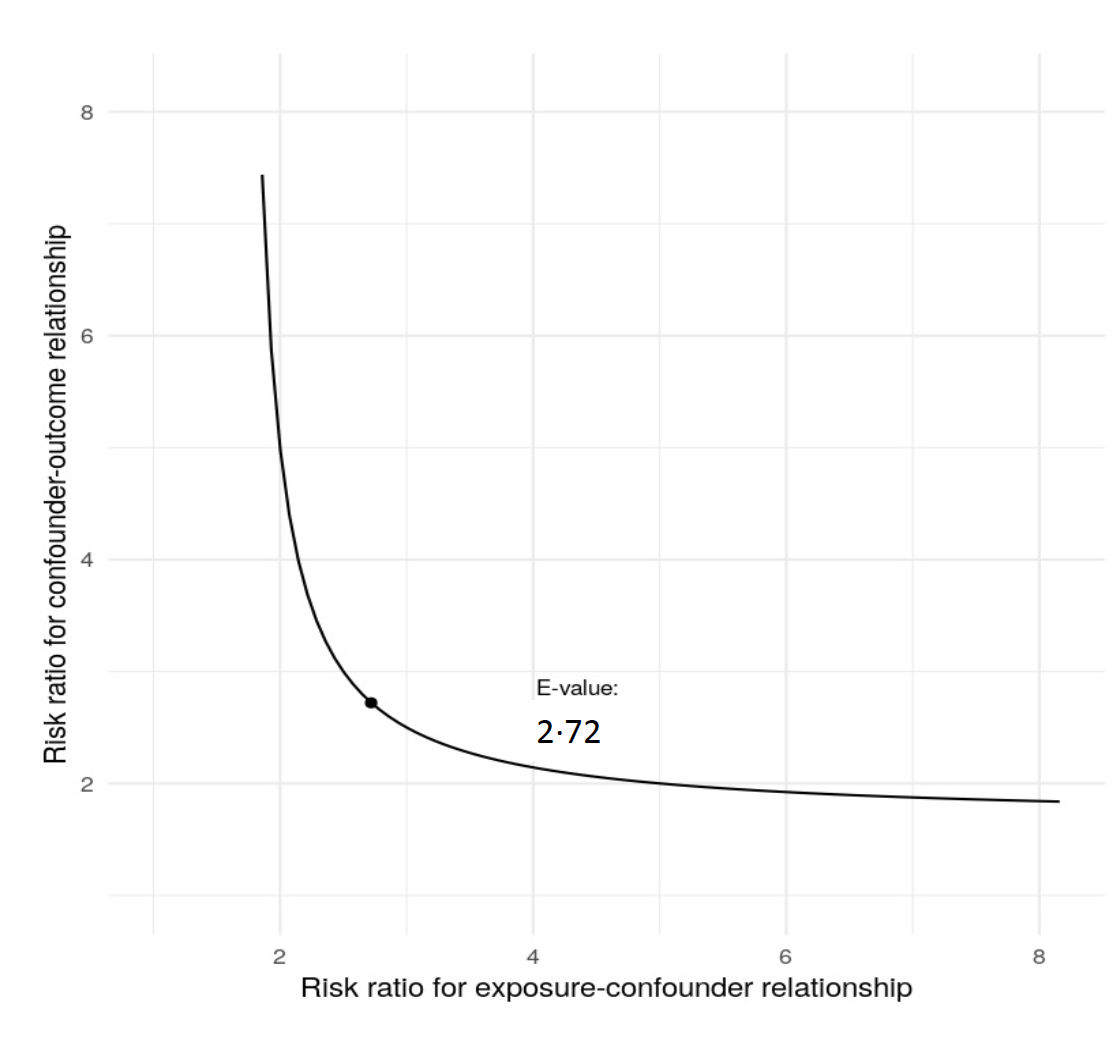


The E-value shows, on the risk ratio scale, the strength of association between the unmeasured confounder and both the exposure and the outcome that would explain away the reported association. Specifically, for the estimated effect of AW-PP on ETI, a risk ratio of 2.72 for both the confounder-outcome relationship and the exposure-confounder relationship would be needed to draw the results to the null value. This shows robustness to potential unmeasured confounding.

## **Figure E5: Risk of intubation between groups in the awake prone position vs. non-awake prone position according to severity of respiratory failure.**


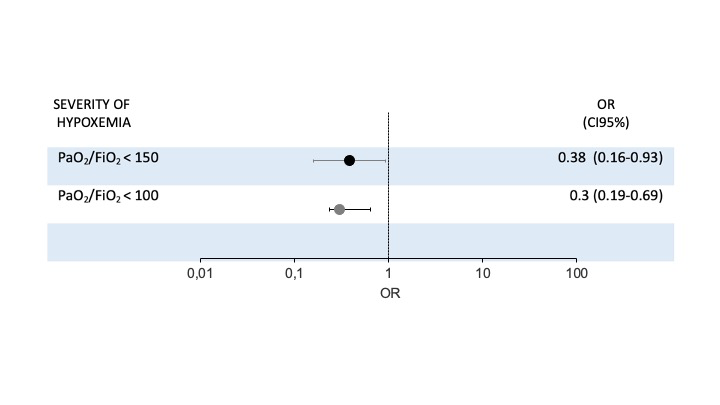


OR indicates odds ratio; 95% CI indicates confidence interval. Non-awake prone positioning group as reference. Abbreviations. AW-PP: awake prone positioning. PaO2/FiO2: ratio of arterial oxygen partial pressure (PaO2) to fractional inspired oxygen (FiO2). Weighted population and adjusted by center and pandemic time.

## **Figure E6: Risk of intubation between groups in the awake prone position vs. non-awake prone position according to predominant body position.**

**
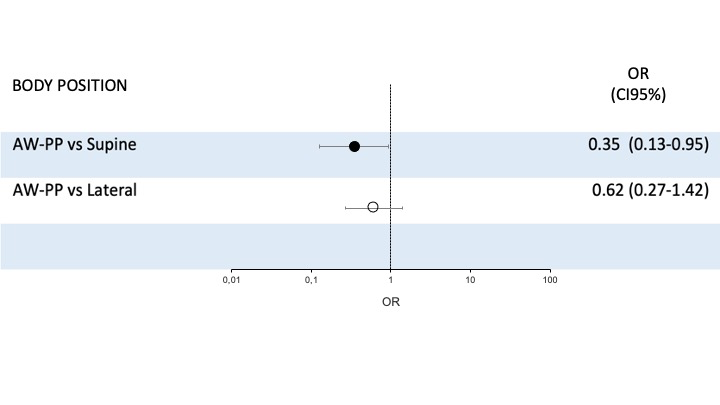
**

OR indicates odds ratio; 95% CI indicates confidence interval. Non-awake prone positioning group (supine or lateral) as reference. Abbreviations. AW-PP: awake prone positioning. Weighted population and adjusted by center and pandemic time.

## **Figure E7: Risk of intubation and risk of death between groups in awake prone position and non-prone position in the overall population study and in the restricted population comparing awake prone position and non-prone position-zero hours in prone.**


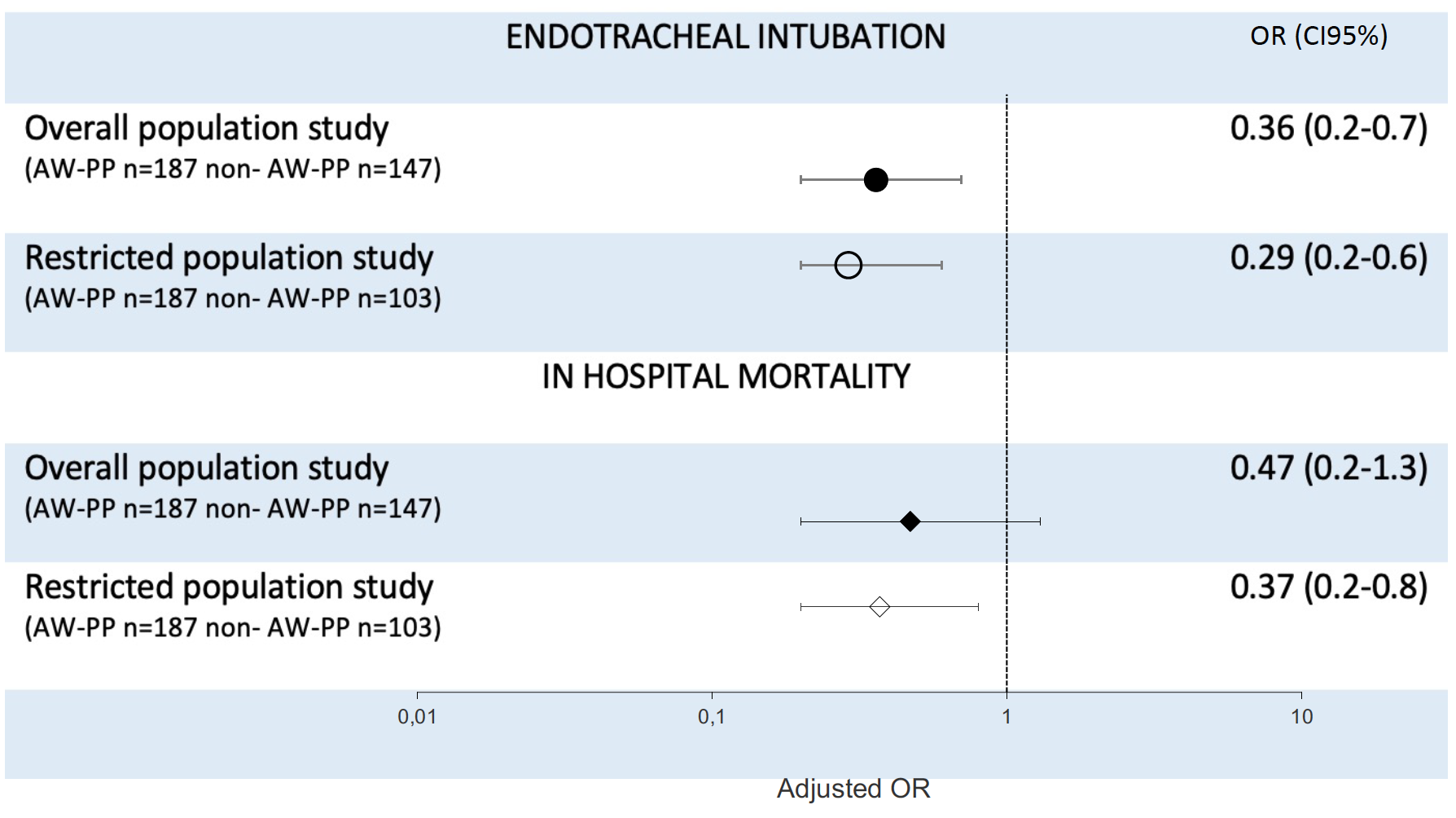


OR indicates odds ratio; 95% CI indicates confidence interval. Non-awake prone positioning group as reference. Abbreviations. AW-PP: awake prone positioning. PaO2/FiO2: ratio of arterial oxygen partial pressure (PaO2) to fractional inspired oxygen (FiO2). Weighted population and adjusted by center and pandemic time.

##

## **Figure E8. Cumulative incidence of endotracheal intubation over time in the study population**


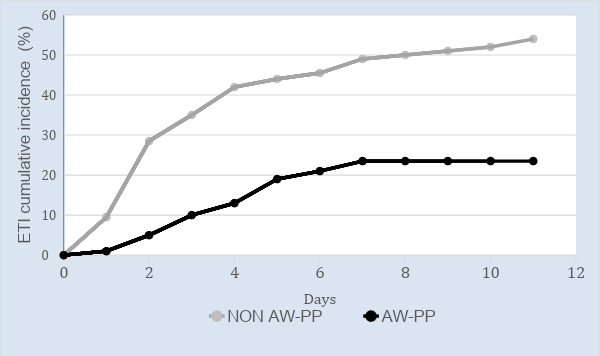


**Days**

NON-PP

Abbreviations. ETI: endotracheal intubation. AW-PP: awake prone positioning

## **Figure E9. Ratio of arterial oxygen partial pressure (PaO_2_) to fractional inspired oxygen (FIO_2_) over the time in awake prone position and non-prone position patients.**

**
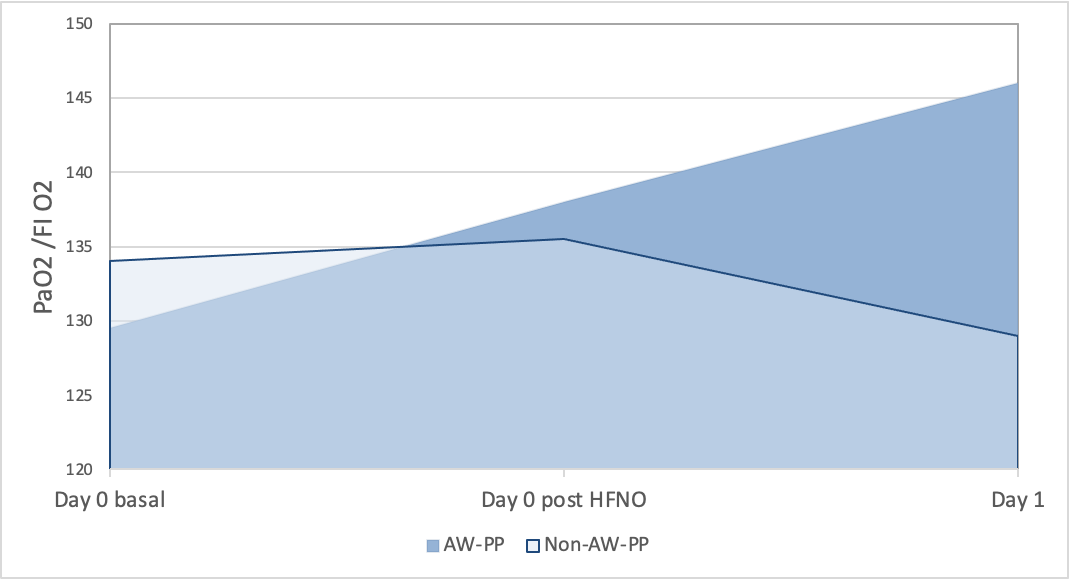
**

**REFERENCES**

[1. Rosenbaum PR, Rubin DB. The central role of the propensity score in observational studies for causal effects. Biometrika. Oxford University Press; 1983;70:41–55.](http://paperpile.com/b/Z8qVnR/9PM1)

[2. Xian Y, O’Brien EC, Fonarow GC, Olson DM, Schwamm LH, Hannah D, et al. Patient-Centered Research into Outcomes Stroke Patients Prefer and Effectiveness Research: Implementing the patient-driven research paradigm to aid decision making in stroke care. Am Heart J. 2015;170:36–45, 45.e1–11.](http://paperpile.com/b/Z8qVnR/Czmp)

[3. Austin PC. The use of propensity score methods with survival or time‐to‐event outcomes: reporting measures of effect similar to those used in randomized experiments [Internet]. Statistics in Medicine. 2014. p. 1242–58. Available from:](http://paperpile.com/b/Z8qVnR/Zp6j) <http://dx.doi.org/10.1002/sim.5984>

[4. Austin PC, Stuart EA. Moving towards best practice when using inverse probability of treatment weighting (IPTW) using the propensity score to estimate causal treatment effects in observational studies. Stat Med. 2015;34:3661–79.](http://paperpile.com/b/Z8qVnR/BMlU)

[5. Austin PC. An Introduction to Propensity Score Methods for Reducing the Effects of Confounding in Observational Studies. Multivariate Behav Res. Taylor & Francis; 2011;46:399–424.](http://paperpile.com/b/Z8qVnR/8apS0)

[6. VanderWeele TJ, Hernán MA, Robins JM. Causal directed acyclic graphs and the direction of unmeasured confounding bias. Epidemiology. 2008;19:720–8.](http://paperpile.com/b/Z8qVnR/UvoPp)

[7. Etminan M, Collins GS, Mansournia MA. Using Causal Diagrams to Improve the Design and Interpretation of Medical Research. Chest. 2020;158:S21–8.](http://paperpile.com/b/Z8qVnR/EKfL5)

[8. Hernán MA, Hernández-Díaz S, Robins JM. A structural approach to selection bias. Epidemiology. 2004;15:615–25.](http://paperpile.com/b/Z8qVnR/zS7pN)

[9. Textor J, van der Zander B, Gilthorpe MS, Liśkiewicz M, Ellison GTH. Robust causal inference using directed acyclic graphs: the R package “dagitty” [Internet]. International Journal of Epidemiology. 2017. p. dyw341. Available from:](http://paperpile.com/b/Z8qVnR/NQXL7) <http://dx.doi.org/10.1093/ije/dyw341>

[10. VanderWeele TJ, Ding P. Sensitivity Analysis in Observational Research: Introducing the E-Value. Ann Intern Med. 2017;167:268–74.](http://paperpile.com/b/Z8qVnR/um3gp)
